# Women’s Experiences of Interstitial Cystitis/Painful Bladder Syndrome

**DOI:** 10.1177/0193945921990730

**Published:** 2021-01-29

**Authors:** Angela Kirkham, Katherine Swainston

**Affiliations:** 1 School of Social Sciences, Humanities & Law, Teesside University, Middlesbrough, UK; 2 Senior Lecturer in Psychology, School of Social Sciences, Humanities & Law, Teesside University, Middlesbrough, UK

**Keywords:** interstitial cystitis, Bladder Pain Syndrome, qualitative methods, lived experience

## Abstract

The aim of this study was to explore the lived experience of interstitial cystitis (IC)/painful bladder syndrome (PBS). A phenomenological approach with emphasis on reflection and openness was adopted. Twenty women diagnosed with IC an average of nine years prior to data collection produced a written account of their experiences. The textual data was analyzed using thematic analysis. Four themes were derived from data analysis: diagnostic uncertainty; restrictions and limitations on life; self-management; and interpersonal relationships and social support. Women reported issues in receiving a diagnosis of IC, undergoing numerous diagnostic tests, and experiencing multiple referrals. Having undergone numerous treatments with limited success, women sought information and management strategies outside of standard medical care and reported a negative impact on sexual and social relationships. The findings illustrate the complex nature of women’s experiences and the physical and psychological impacts and effects of IC/PBS on women’s daily lives.

Interstitial Cystitis (IC), a term often used interchangeably with Bladder Pain Syndrome (BPS) or Painful Bladder Syndrome (PBS), is a condition with a defining characteristic of recurrent pain in the bladder and pelvis, with one or more symptoms including an urgent and frequent need to urinate during the day and at night (National Health Service, 2019; Payne et al., 2009). It is pain that distinguishes IC from conditions such as overactive bladder syndrome. IC is most frequently diagnosed in women (Berry et al., 2011), with estimates of prevalence suggesting that 45 per 100,000 females are diagnosed with IC and 8 per 100,000 men (Patnaik et al., 2017). IC is recognized as a long-term condition, and while some treatments may be effective at reducing pain and symptoms, and some individuals may go into a period of remission, there is no universally accepted cure (Hanno et al., 2015; National Health Service, 2019).

The predominance of psychological research in this field focuses on the negative impact of symptoms on employment status (Beckett et al., 2014), anxiety and depression (McKernan et al., 2018), sexual functioning (Bogart et al., 2011; Liu et al., 2014), and assessing quality of life (Michael et al., 2000). Furthermore, after adjusting for comorbidities and age, individuals with IC score significantly lower on quality of life in dimensions including bodily pain, vitality levels, social functioning, role limitations, and physical functioning than healthy female controls (Michael et al., 2000). Similarly, lower quality of life scores in comparison to the general population were noted by Tripp et al. (2009) who reported that pain, a tendency to worry or catastrophize over symptoms, depression, and a perceived inability to cope were strongly associated with decreased quality of life scores in individuals with IC. Approximately 35% of individuals with IC reported experiencing depression and 52% had experienced a panic attack within the last three months (Watkins et al., 2011). It is argued that there is still insufficient mental health support for IC patients, and that individuals should be supported to prevent increased physical and mental suffering due to maladaptive pain cognitions such as catastrophizing (Muere et al., 2018).

IC has been reported as having a negative impact on sexual functioning and interpersonal relationships (Azevedo et al., 2005). This focus of the current literature on the sexual effects of IC may, however, cause other areas of impact on individuals’ lives to be underrepresented. Accordingly, while some of the impacts of IC are known, there remains a paucity of research, particularly of a qualitative nature, examining the psychological effects of this condition. To address this, the current research explores the lived experience of IC with a focus on, rather than quantifying impacts, enabling individuals to tell their story of living with IC.

## Method

### Design

Participants produced a written account describing their experiences of living with IC, in accordance with a phenomenological approach (van Manen, 1990). The use of an open-ended method of data collection enabled participants to have the freedom to anonymously describe their lived experience of IC, without direction. Interviews are subject to researcher influence and can lead to participants not fully disclosing or remembering information (Braun & Clarke, 2013) as they are required to give an immediate answer (Smith et al., 2009). The use of written accounts meant that participants could choose aspects of their lived experience which were most salient and meaningful to them. Accordingly, the participant-generated written accounts adopted in this study should have increased the dependability of the current data.

### Sample

A purposive sample of 20 women aged over 18 years (age range 21–69 years; mean age 46 years) was recruited via specialist IC online messaging forums. Inclusion criteria were that participants had obtained a medical diagnosis of IC, were over the age of 18 years, and were able and willing to give informed consent. The recruitment adverts were posted free of charge with written permission from all forum administrators being obtained prior to placing the advertisements. Respondents were predominantly from the United Kingdom (n = 9) and USA (n = 11). Individuals interested in participating contacted the researchers for further study details, at which point the inclusion/exclusion criteria were discussed and participants were enrolled into the study if appropriate. All participants self-reported that they had received a diagnosis between 1 year and 36 years ago (approximate mean years since diagnosis = 9, median years since diagnosis = 5). The sample size enabled in depth analysis of women’s experiences to be undertaken while engaging in reflexivity, ensuring transparency, and data saturation.

### Data Collection Methods

Ethical approval was obtained from Teesside University School of Social Sciences, Humanities & Law Research Ethics Sub Committee. Participants who responded to the online post were sent a participant information sheet and were informed that due to the online nature of the study, by submitting their written accounts to the lead researcher (AK), they were giving informed consent and they were informed of their right to withdraw their data at any point up until data analysis. Participants were requested to e-mail a written account of their experience of living with IC (and their age, location, and years since diagnosis, if they wished) to the first researcher. Participants were asked to “Please describe your experience of IC.” No prompts were given. Participants had eight weeks to write their account and return this to the lead researcher (AK). Upon receipt of a written account, a debrief sheet providing details of organizations providing IC-related support was emailed to the participant. A reflexive journal was kept by the first researcher throughout the research process and by the second researcher during data analysis. Thematic analysis is based upon the premise of researchers as active and reflexive (Braun & Clarke, 2019) and so these reflections were discussed during the data analysis process.

### Data Analysis

The textual data was analyzed using thematic analysis (Braun & Clarke, 2006) in order to identify patterns of data and develop themes and sub-themes to represent participants’ experiences of IC. Six steps of analysis were followed, beginning with the authors familiarizing themselves with the data, generating initial codes and searching for themes. Themes were reviewed, defined, and named, and the final paper was produced. The lead researcher has personal experience of IC and, accordingly, analysis was undertaken by both authors individually and agreements were reached regarding theme content and theme names. All participants have been allocated a pseudonym.

## Results

Four core themes were identified from the data analysis: diagnostic uncertainty; restrictions and limitations on life; self-management; and interpersonal relationships and social support. Each theme comprises inter-related sub-themes that highlight the specific issues encompassed within the theme. All themes are inter-related and reflect participants stories about living with IC.

### Diagnostic Uncertainty

Participants expressed feelings of frustration with their experience of diagnosis, and they typically described a lengthy process of being re-referred, re-tested, and not being believed that their condition had a physical cause. Two key issues were evident constituting sub-themes of diagnostic delay and increasing frustration.

#### Diagnostic delay

Seeing multiple health care professionals during the search for a diagnosis was commonly reported by participants and this contributed to delays from first clinical presentation to diagnosis.

I was first diagnosed with IC about three years ago, but I have been suffering from the condition for about twelve years. I have seen numerous medical professionals over that time, who generally gave me antibiotics for cystitis, or told me there was nothing wrong, as evidenced by scans. (Bonnie)

Louise reported a similar experience:

Much to my dismay, I travelled to numerous doctors and received many different diagnoses prior to discovering the IC on my own. I was told I was crazy and that these symptoms were all in my head and that a psychiatrist was what I needed. I received my nursing magazine in the mail. There was an article about IC and it’s symptoms and medical management. I was so frustrated, in so much pain and discomfort on a daily basis. I took the medical article to my gynecologist who recommended a urologist. My scope revealed IC with glomerulations and Hunners ulcers. A classic IC patient.

The prolonged experience of diagnostic uncertainty and seeing multiple health care professionals from differing specialties in the search for a diagnosis led to increasing frustration.

#### Increasing frustration

The impact of feeling that symptoms were dismissed or not believed alongside experiencing debilitating symptoms had an impact on participants psychological health.

My bladder problems began about 18 years ago. My GP was at a loss as to what might be causing the problem and referred me, at my insistence, to an urologist. I had a cystoscopy but my bladder looked perfectly healthy. I was then told I must have a gynecological problem. By this time I was unable to work, I was permanently exhausted and tearful—I knew I was in pain and couldn’t understand why no-one could find the cause. I asked for a second opinion. I was later referred me to a professor of urology and after a further cystoscopy and biopsy he diagnosed interstitial cystitis. At last someone believed me—I wasn’t going mad!. (Riaan)

Riaan’s account of the diagnostic process is representative within this sample and highlights a frustration experienced by women with IC who are struggling to receive a diagnosis. This quote also demonstrates the relief described by women upon finally receiving a diagnosis after, in most cases, many years of unexplained symptoms. A general frustration around understanding of IC in addition to diagnosis was equally apparent, given the resulting impact on women’s lives.

I have lived with IC for 5 years and it is not getting any easier. The lack of knowledge and understanding of this condition is almost beyond comprehensible in this day and age when it is such a debilitating, excruciatingly painful, and unsociable condition. (Rebecca)

### Restrictions and Limitations on Life

Participants described how the uncertainty of the onset and presentation of symptoms during an episode of IC had a restricting impact on activities of daily living. Women reported having to abandon enjoyable activities, losing the life they had before IC, an inability to perform daily tasks without pain, and the impact this had on decision-making and particularly making choices about the future. Two sub-themes depict these inter-linked issues: losing a normal life and impact on activities of daily living.

#### The loss of a normal life

Participants mourned the life they had before IC and felt a loss for the life they should be living.

My life is severely debilitating and indeed very difficult to cope with. My complete life has been taken over by this condition. . .even basic tasks have become a nightmare to complete. (Patty)

Participants abandoned activities that they previously found to be enjoyable, and activities, plans, and commitments often needed to be changed given the presence and severity of IC symptoms.

Interstitial Cystitis is so constant and restricting in my life that there are still many things I do not do and IC is what my life unfortunately revolves around. . .. I feel like my life hadn’t even had a chance to begin and now unfortunately I am limited by IC. I mourn the life that I thought I would have before getting IC. (Lucy)

Lucy’s account suggests that IC not only limits her life but also restricts her options for the future. Similarly, the impact of IC on restricting quality of life is evidenced within Sandra’s written account:

IC can make you want to give up, it’s not only the physical but the emotional and mental toll. . .you cannot even imagine how it feels to urinate 50 plus time a day or urinate and 5 minutes later have the urge to go again. . .it is worse than any pain because it does not go away. Your quality of life is impacted in ways you can’t imagine. (Sandra)

Adjustment and learning to live with the challenges of IC were central to women’s accounts and brought about a change to women’s sense of self. Yet, they hoped for a cure and a chance to live the life their life without pain.

The most complicated aspect of living with IC for me has been acknowledging that it can be debilitating and having to make huge lifestyle adjustments that ultimately changed who I am and what I can pursue. (Sophie)

I wait in hope as all of us do for something which will cure this horrible disease and give me back my life. (Riaan)

#### Impact on activities of daily living

All women wrote extensively about the impact of IC on their day to day lives. Restrictions within life included everyday activities, social interactions, and employment situations, with some participants describing having given up work due to ongoing symptoms and difficulties in managing IC within the workplace.

Getting up at night to pass urine could be as many as 10 times or more and every hour during the day. This led to sleep deprivation which led to me making mistakes and being forgetful in work. I have had to stop working due to the fact that I could not cope with working on so little sleep. (Emily)

Participants stated that, due to the pain, sleep-deprivation, frequency of urination, and other IC symptoms, they are unable to perform everyday tasks or activities. This has led to restrictions and limitations in their leisure time, and at work.

My quality of life has been badly affected due to the constant pain and dreadful feeling of needing to urinate. This has resulted in nearly a year off work, reduced working hours on return, unable to sleep at least 2 nights a week, cancelled holidays, and giving up my hobby. (June)

In some cases the result was a loss of employment or hobby. Individuals’ accounts illustrate the many and varied difficulties experienced when living with IC. An impact on women’s mental health was apparent given the restrictions and adjustments necessary to manage the condition.

Whenever I was in any kind of situation. . .where it would be noticeable if I went more than once in 30 minutes I had such extreme anxiety that I was going to have an accident. I ended up being housebound for months and extremely anxious; even at the thought of someone coming to the door, having a conversation and me needing to go to the toilet during a conversation. I became very depressed and eventually saw a psychiatrist who prescribed me antidepressants for the first time. (Lucy)

### Self-management

Dissatisfaction with treatment options and a reluctance to undergo some potential treatments for IC had resulted in women seeking and attempting self-management strategies. This theme discusses two sub-themes: researching options and trial and error. Women researched their options often after undergoing numerous medical tests and trying several medical interventions that had been unsuccessful in reducing pain.

#### Researching options

Several participants described actively researching IC to ensure that they were informed about the latest perspectives and developments in the field. For some participants, researching IC resulted in them not wanting to undergo medical interventions and attempting to direct and have control over their treatment plan.

They [antibiotics] didn’t help and my pain got worse. The urologist said that I would need to be scoped; however, she wanted to do a hydrodistention as well. I had been researching IC constantly up to this point, and knew that hydrodistention or even the scope was no longer recommended. The anecdotal evidence from the ICN forums made it seem like there was a 50/50 chance for the hydrodistention to make the pain worse. I had even brought the papers to show the urologist, but she didn’t look at them or listen to me. I cried in the office, not wanting those procedures. (Juliet)

While Juliet’s experience of presenting research to the medical profession was not a positive one, other women described the benefits of being pro-active in doing so.

I started searching on Facebook to find some groups with IC people, I read and read and read what was helping women and what wasn’t. By my next Urology appointment I told my doc which meds I wanted to try and she agreed. My doc calls me her best IC patient due to all the knowledge I found on my own. (Alexandra)

As a result of conducting their own research, some women had chosen to move away from mainstream medical treatments including antibiotics and to instead adopt dietary and lifestyle changes. Listening to their bodies and focusing on maximizing general well-being and reducing stress were common statements throughout women’s written accounts.

I also try to listen to my body more, focus on getting enough quality sleep, saying “NO” to additional stressors, not working out too much and not being so hard on myself. I need to get my body from an inflamed hyper alert state to a calmer, more relaxed happy state that is conducive to healing. (Dani).

#### Trial and error

Narratives around medications and medical and non-clinical interventions were apparent in all participants accounts. Antibiotics, pain medication, anti-inflammatory drugs, anti-depressants, surgery, bladder installations, physical therapy, and pelvic floor exercises were frequently reported. Always carrying “pee pots, pads, wipes” (Rebecca), dietary regulation, dietary supplements, the use of ice packs, stress reduction techniques, Botox injections into the bladder, and scoping out bathroom availability were strategies documented for managing IC. Adopting a combination of medical and self-management strategies was most evident, with bladder installations in association with lifestyle changes being described by several participants as providing relief during flare-up’s.

Fast forward to 1 year ago, my flares started increasing in intensity and length. I contacted a urologist, she called it Interstitial Cystitis. IC, I finally had a name for this! I am taking bladder instillations twice a week now. I am cautiously optimistic this is working for me right now. I am also trying the IC diet, and reading forums, and finding out I am not alone! (Emma)

Emma’s quotation highlights how medical intervention, nutritional guidance, and support from others can be of benefit to women experiencing IC as well as the importance of diagnostic certainty.

### Interpersonal Relationships and Social Support

The impact of IC on interpersonal and intimate relationships and the support provided (or not) via these relationships was central to women’s accounts of living with IC. Women wrote about painful experiences during sexual intercourse and the loss of intimacy within sexual relationships as a result of the symptoms of IC as well as the understanding of others about their condition and the limitations it brought.

#### Intimacy and sexual relationships

The impact on sexual relationships was evident in women’s accounts. For some women sexual intercourse was too painful and this had an impact on the sense of womanhood.

Sex is off the agenda most of the time which diminishes me as a woman. (Sylvia)

Miranda perceived her relationship with her partner to have suffered as a direct result of IC.

In the last five years or so we haven’t had intercourse largely due to the IC. I’m really sorry for my husband, but the pain wasn’t usually worth the pleasure for me, and he was aware of that often. It’s partly affected our closeness, and that’s really sad. People used to tell us to not be so “lovey dovey” because we were always so tied to each other, and now not so much. (Miranda)

For some participants, IC was described as resulting in the loss of relationships.

The man I was dating could no longer take being with me since he could not sleep with me or deal with the constant urination and pain. He was very nice to me but he said he felt helpless. He wanted to travel and go on weekend outings and I am unable to because of the pain and constant need to urinate. (Gillian)

The intimacy and closeness to partners within sexual relationships was undoubtedly perceived to have been affected by IC, and this had an emotional impact on women in this study.

#### Understanding among friends

Women described perceiving others to not fully understand or appreciate the limitations that are a result of IC.

Family and friends have a hard time understanding because they have healthy normal bladders and don’t understand why it just can’t be fixed!! It is a struggle every day. (Sandra)

Similarly, Mel notes how her friendship group became smaller as a result of her symptoms:

Some friends and even family members didn’t understand why going to the bathroom a lot and having bladder pain was such a big deal. Outwardly, I got dressed, put makeup on, went to school (often in terrible pain), and did my job, interacted with others—no visible sign of trouble—so many did not get it. Over time, my circle of friends got smaller—for example, I would have to cancel a lunch date or a weekend trip with my husband and friends at the last minute because I was having a bad flare up. I guess over time—that gets to be old stuff no one wants to hear. (Mel)

Some women did not discuss IC with others, and only a few close family/friends were aware of their diagnosis. Embarrassment and humiliation were words frequently found within women’s accounts of IC and difficulties talking about bladder issues were reported. For some modesty, privacy concerns and fears of urological and gynaecological examinations had an impact on not only social interactions with those close to them but also with health care professionals.

I know I have isolated myself but the embarrassment of having to discuss this situation is too much. I went to counselling (my doctors idea) last year where I was encouraged to be more open about my condition, most people didn’t know I was ill. (Amelia)

The invisibility of IC and the symptoms not being fully understood by others was perceived to slowly contribute to women’s social circles becoming reduced. Linked to the previous theme of self-management, participants sought support from online forums comprised of others experiencing IC and these were noted to be a valuable source of information as well as understanding.

The online network was really the lifesaver for me. They educated me about the illness and offered so many resources. I’ve been able to find things specific to IC needs, like cushions & perineum ice packs that you can’t find anywhere. Through their online forums I saw I wasn’t alone, I had the same questions as others and that you can have a “normal” life even with chronic pain. (Diana)

## Discussion

A key finding from the present study is that participants described experiencing issues during the diagnostic process, having to undergo multiple tests with various clinicians, before receiving their diagnosis of IC. This diagnostic uncertainty often resulted in women actively seeking information for themselves and presenting this to health care professionals in an attempt to gain control over their condition and treatment. As such, it may be reasonable to question whether the variation and contestation surrounding the definitions and diagnostics of IC (Fall & Peeker, 2015) could play a role in the diagnostic issues women describe. The data suggests that many participants found the process of being diagnosed with IC time-consuming and frustrating, which may be due to the many different clinical criteria and diagnostic tools used for this illness (Fall & Peeker, 2015).

Positive doctor-patient communication, and effective information provision, have been strongly linked with factors including patient satisfaction, ability to cope with a new diagnosis of illness, and health outcomes **(**Riedl & Schüßler, 2017), yet are issues highlighted in women’s accounts of their experiences of IC. Indeed, uncertainty about an individual’s illness (relating to delayed diagnosis, or poor information provision) can be related to poor psychosocial adjustment and worse symptoms (Brown et al., 2020). These findings suggest that improvements may need to be made in providing individuals with relevant information and advice following their IC diagnosis; and that this may improve the individual’s ability to cope with their condition, and in-turn improve their overall QOL.

Elements of the themes that emerged from participant’s written accounts particularly within the theme “Restrictions and limitations on life” reflect issues previously identified within the IC literature; such as the effect of IC on an individual’s ability to work (Beckett et al., 2014; Vasudevan & Moldwin, 2017) and mental well-being (Watkins et al., 2011). The current findings support the existence of a connection between IC and mental health conditions; suggesting that individuals with IC may benefit from increased access to psychological treatments, as suggested by Watkins et al. (2011). While previous quantitative work has struggled to attribute causality to the mental health-IC correlation (Beckett et al., 2014; Watkins et al., 2011), the women in the present study perceived IC to be a cause of their depression, anxiety, and overall decreased mental well-being. Although there is no standard approach to treatment for the physical symptoms of IC, the work of Muere and colleagues (2018) indicates that physical pain is exacerbated by psychological distress such as depression, anxiety, and catastrophizing.

Women often described undertaking self-management strategies including changing their diet and trying to minimize stressful situations, but the information on which these changes were based was largely gained from Internet searches and online support groups rather than from health care professionals. The narratives included mention of trying numerous medical interventions, some with limited success, but more in-depth exploration of the discussions had around strategies for support during clinical consultations would be beneficial. What is evident is that some women were wary of discussing IC yet actively searched for understanding and support, which suggests that potential improvements are needed in information provision for women with IC. The women in this study appeared to take on the role of “expert” when making treatment decisions, in part due to perceptions that medical professionals lacked knowledge to help them. While not in relation to IC this has been identified previously with a negative psychological impact being reported for patients taking on this role (Budych et al., 2012).

The negative impact of IC on sexual function is well reported and researched using quantitative methodologies designed to assess quality of life and sexual functioning (Azevedo et al., 2005; Tripp et al., 2009). Therefore, participants’ decision to cite sexual function in this current study, without being directed to do so, indicates that these factors are perceived to be salient and of importance in the lived experiences of individuals with IC. Intimacy and sexual relationships are, however, only part of women’s story regarding interpersonal relationships and support. Understanding among friends shows the importance of social support and the impact of feeling unsupported and unable to discuss symptoms, often due to embarrassment and feelings of shame at discussing a personal and intimate condition. For some women such feelings combined with seeing multiple medical professionals in the search for diagnosis and perceiving that they were not being believed led to delays in help-seeking and a lack of trust of health care professionals. The fear of urological and gynecological examination and invasive diagnostics and treatment further limited some women’s willingness to seek medical support and undergo required procedures.

The present study is the first of its kind to explore the lived experience of IC utilizing a written narrative as the unit of analysis and adds to the limited research of a qualitative nature exploring women’s experiences of IC. One methodological limitation of the present study is the reliance on retrospective participant narratives, as such data may be subject to recall-bias and the data may represent subjectively-salient endpoints of a specific experience. Similarly, it is possible that participants who responded to the recruitment call were experiencing more disease concern, anxiety, and treatment issues. However, a broad sample of participants, in terms of age and time since diagnosis, were recruited and patterns within the data emerged across the sample as a whole. That said, this sample of 20 participants may not be representative of other individuals experiences of IC and differences in health care systems may influence experiences of the diagnostic and treatment processes. Issues around the accuracy of the diagnosis of IC may is also a limitation in itself. Furthermore, while the vast majority of individuals with IC are female, males can be diagnosed with IC yet are not represented in the current study. Exclusions were not in place in terms of gender, but the study received only female respondents. Future research could examine male experiences of IC and explore experiences from the point of diagnosis utilizing a longitudinal methodology.

Clinical research suggests risk of false IC diagnosis and that some potentially curable cases of UTI may go undetected and untreated due to diagnostic issues although some clinicians have developed alternative UTI testing methods, which shows promise (Swamy et al., 2018). It is therefore plausible that some of the women in this study may have a condition other than IC. There is, as yet, no existing cure for IC, and the narratives in this study highlight that there are psychological effects of living with this illness in relation to sexual function, interpersonal relationships, ability to work, and mental well-being. Improving the experience of diagnosis and treatment as well as considering the psychological well-being of individuals with IC warrants further study to identify whether psychological support for the pervasive psychological, social, and sexual difficulties described by those living with IC may be beneficial.

To conclude, the purpose of this research was to gain an understanding of the lived experience of individuals with IC. Thematic data analysis uncovered common themes of participant experiences revealing that women were psychologically affected by IC, as their condition restricted many aspects of their life. Although further investigation is warranted, the findings of this research could begin to inform health care professionals about the challenges that individuals with IC face in their daily living, and how their quality of life is affected.

## References

[bibr1-0193945921990730] Azevedo K. Nguyen A. Rowhani-Rahbar A. Rose A. Sirinian E. Thotakura A. Christopher K. Payne M. D. (2005). Pain impacts sexual functioning among interstitial cystitis patients. Sexuality and Disability, 23, 189–208. 10.1007/s11195-005-8927-y

[bibr2-0193945921990730] Beckett M. K. Elliot M. N. Clemens J. Q. Ewing B. Berry S. H. (2014). Consequences of Interstitial cystitis/bladder pain symptoms on women’s work participation and income: Results from a national household sample. The Journal of Urology, 191, 83–88. 10.1016/j.juro.2013.07.018 23872030 PMC4085039

[bibr3-0193945921990730] Berry S. H. Elliot M. N. Suttorp M. Bogart L. M. Stoto M. A. Eggers P. Nyberg L. Clemens Q. (2011). Prevalence of symptoms of bladder pain syndrome/interstitial cystitis among adult females in the United States. The Journal of Urology, 186, 540–544. 10.1016/j.juro.2011.03.132 21683389 PMC3513327

[bibr4-0193945921990730] Bogart L. M. Suttorp M. J. Elliott M. N. Clemens J. Q. Berry S. H. (2011). Prevalence and correlates of sexual dysfunction among women with bladder pain syndrome/interstitial cystitis. Urology, 77, 576–580. 21215432 10.1016/j.urology.2010.10.016 PMC3059214

[bibr5-0193945921990730] Braun V. Clarke V. (2006). Using thematic analysis in psychology. Qualitative Research in Psychology, 3(2), 77–101. 10.1191/1478088706qp063oa

[bibr6-0193945921990730] Braun V. Clarke V. (2013). Successful qualitative research. SAGE Publications.

[bibr7-0193945921990730] Braun V. Clarke V. (2019). Reflecting on reflexive thematic analysis. Qualitative Research in Sport, Exercise & Health, 11(4), 589-597. 10.1080/2159676X.2019.1628806

[bibr8-0193945921990730] Brown A. Hayden S. Klingman K. Hussey L. C. (2020). Managing uncertainty in chronic illness from patient perspectives. Journal of Excellence in Nursing & Healthcare Practice, 2(1), 1–16. 10.5590/JENHP.2020.2.1.01

[bibr9-0193945921990730] Budych K. Helms T. M. Schultz C. (2012). How do patients with rare diseases experience the medical encounter? Exploring role behavior and its impact on patient-physician interaction. Health Policy, 105, 154–164. 10.1016/j.healthpol.2012.02.018 22464590

[bibr10-0193945921990730] Fall M. Peeker R. (2015). Classic interstitial cystitis: Unrelated to BPS. Current Bladder Dsyfunction Reports, 10, 95–102.

[bibr11-0193945921990730] Hanno P. M. Erickson D. Moldwin R. Faraday M. M. (2015). Diagnosis and Treatment of Interstitial Cystitis/Bladder Pain Syndrome: AUA Guideline Amendment. The Journal of Urology, 193(5), 1545–1553. 10.1016/j.juro.2015.01.086 25623737

[bibr12-0193945921990730] Liu B. Su M. Zhan H. Yang F. Li W. Zhou X. (2014). Adding a sexual dysfunction domain to UPOINT system improves association with symptoms in women with interstitial cystitis and bladder pain syndrome. Urology, 84, 1308–1313. 25312548 10.1016/j.urology.2014.08.018

[bibr13-0193945921990730] McKernan L. C. Walsh C. G. Reynolds W. S. Crofford L. J. Dmochowski R. R. Williams D. A. (2018). Psychosocial co-morbidities in Interstitial Cystitis/Bladder Pain syndrome (IC/BPS): A systematic review. Neurourology and Urodynamics, 37, 926–941. 10.1002/nau.23421 28990698 PMC6040587

[bibr14-0193945921990730] Michael Y. L. Kawachi I. Stampfer M. J. Colditz G. A. Curhan G. C. (2000). Quality of life among women with interstitial cystitis. The Journal of Urology, 164, 423–427. 10.1016/S0022-5347(05)67376-4 10893601

[bibr15-0193945921990730] Muere A. Tripp D. A. Nickel J. C. Kelly K. L. Mayer R. Pontari M. Moldwin R. Carr L. K. Ynag C. C. Nordling J. (2018). Depression and coping behaviors are key factors in understanding pain in interstitial cystitis/bladder pain syndrome. Pain Management Nursing, 19, 497–505. 10.1016/j.pmn.2017.11.001 29501360

[bibr16-0193945921990730] National Health Service. (2019). Interstitial cystitis. https://www.nhs.uk/conditions/interstitial-cystitis/

[bibr17-0193945921990730] Patnaik S. S. Lagana A. S. Vitale S. G. Butice S. Noventa M. Gizzo S. Valenti G. Rapisarda A. M. C. La Rosa V. L. Magno C. Triolo O. Dandolu V. (2017). Etiology, pathophysiology, and biomarkers of interstitial cystitis/painful bladder syndrome. Archives of Gynecology & Obstetrics, 295, 1341–1659. 10.1007/s00404-017-4364-2 28391486

[bibr18-0193945921990730] Payne R. A. O’Connor R. C. Kressin M. Guralnick M. L. (2009). Endoscopic ablation of Hunner’s lesions in interstitial cystitis patients. Canadian Urological Association Journal, 3, 473–477. 20019976 10.5489/cuaj.1178 PMC2792435

[bibr19-0193945921990730] Riedl D. Schüßler G. (2017). The influence of doctor-patient communication on health outcomes: A systematic review. Zeitschrift für Psychosomatische Medizin und Psychotherapie, 63(2), 131–150. 28585507 10.13109/zptm.2017.63.2.131

[bibr20-0193945921990730] Smith J. A. Flowers P. Larkin M. (2009). Interpretative phenomenological analysis: Theory, method and research. SAGE Publications.

[bibr21-0193945921990730] Swamy S. Barcella W. De Iorio M. Gill K. Khasriya R. Kupelian A. S. Rohn J. L. Malone-Lee J. (2018). Recalcitrant chronic bladder pain and recurrent cystitis but negative urinalysis: What should we do? International Urogynecology Journal, 29, 1035–1043. 29556674 10.1007/s00192-018-3569-7 PMC6004281

[bibr22-0193945921990730] Tripp D. A. Nickel J. C. Fitzgerald M. P. Mayer R. Stechyson N. Hsieh A. (2009). Sexual functioning, catastrophising, depression, and pain, as predictors of quality of life in women with interstitial cystitis/painful bladder syndrome. Female Urology, 73, 987–992. 10.1016/j.urology.2008.11.049 19394494

[bibr23-0193945921990730] van Manen M. (1990). Researching lived experience. State University of New York Press.

[bibr24-0193945921990730] Vasudevan V. Moldwin R. (2017). Addressing quality of life in the patient with interstitial cystitis/bladder pain syndrome. Asian Journal of Urology, 4, 50–54. 10.1016/j.ajur.2016.08.014 29264207 PMC5730899

[bibr25-0193945921990730] Watkins K. E. Eberhart N. Hilton L. Suttorp M. J. Hepner K. A. Clemens J. Q. Berry S. H. (2011). Depressive disorders and panic attacks in women with bladder pain syndrome/interstitial cystitis: A population-based sample. General Hospital Psychiatry, 33, 143–149. 10.1016/j.genhosppsych.2011.01.004 21596207 PMC3099040

